# Magnifying Chromoendoscopy with Flexible Spectral Imaging Color Enhancement, Indigo Carmine, and Crystal Violet in Predicting the Histopathology of Colorectal Polyps: Diagnostic Value in a Scare-Setting Resource

**DOI:** 10.1155/2022/6402904

**Published:** 2022-07-13

**Authors:** Nguyen Binh Pham, Khanh Truong Vu, Nam Hoai Nguyen, Ha Thi-Ngoc Doan, Thanh Trung Tran

**Affiliations:** ^1^Gastroenterology & Hepatology Center, Bach Mai Hospital, Hanoi, Vietnam; ^2^Ha Noi Medical University, Hanoi, Vietnam; ^3^National Institute of Hygiene and Epidemiology, Hanoi, Vietnam

## Abstract

**Background and Aims:**

Virtual magnifying chromoendoscopy with flexible spectral imaging color enhancement (FICE), image-enhanced endoscopy techniques, and dye-staining magnifying chromoendoscopy (with Indigo carmine and Crystal violet) have contributed to better visualization of the pit pattern and vascular structure of colorectal polyp. Therefore, magnifying chromoendoscopy is capable of predicting the histopathological results of colorectal polyp without biopsy and remains their diagnostic values over time, especially in scare-setting resources. This study compared the images of magnifying chromoendoscopy between FICE, Indigo carmine, and Crystal violet and then assessed their diagnostic values based on colorectal polyps' histopathology as a gold standard.

**Methods:**

A total of 332 polyps of 266 patients were endoscopically evaluated from June 2016 to September 2019. After identified by white light endoscopy, polyps continued to be evaluated by virtual magnifying chromoendoscopy (×50-150 times) with FICE. The capillary-vessel pattern was divided into 5 subtypes according to the number, morphology, and distribution of the fine blood vessels according to Teixeira classification. Next, they were stained with Indigo carmine 0.2% and then Crystal violet 0.05% and were classified according to Kudo's pit pattern classification. Finally, polyps were resected by endoscopy or surgery and biopsy and compared with histopathological results of either neoplastic or nonplastic polyp.

**Results:**

The number of neoplastic polyps was 278/332 with 231 adenoma polyps and 47 carcinoma polyps. Magnifying chromoendoscopy has high sensitivity and accuracy when compared with the histopathological results of colorectal polyps. The sensitivity, specificity, and accuracy of magnifying chromoendoscopy with Crystal violet are 97.2%, 72.2%, and 93.0%; with Indigo carmine are 96.0%, 72.2%, and 92.1%; and with FICE are 92.1%, 68.5%, and 88.3%.

**Conclusions:**

Among the three methods, Crystal violet has the highest sensitivity and accuracy in predicting histopathological results of colorectal polyps. FICE has shown its diagnostic value with reliable sensitivity and accuracy and should still be a reasonable endoscopic choice for physicians in scare-setting resources regardless its moderate specificity. Physicians should base on their facility and capability to determine an appropriate endoscopy technique.

## 1. Introduction

Colorectal polyps are a gastrointestinal disease caused by an overgrowth of the mucosa and submucosa of the colon. In Asia and Europe, the prevalence of colorectal polyps is up to 12% and 26% of the population, and the pathogenesis of the disease is strongly related to factors such as lifestyle, sex, age, and genetics [1, 2]. Polyps are divided into two main groups: neoplastic polyps (tubular adenoma, villous adenoma, tubulovillous adenoma, and sessile serrated polyps) and nonneoplastic polyps (inflammatory polyps, hyperplastic polyps, juvenile polyps, and hamartomatous polyps) [2]. In particular, neoplastic polyps have a high risk of progressing to colorectal cancer. According to Silva et al., 60%-90% of colorectal cancers have progressed from colorectal polyps [3]. Currently, endoscopy is the most effective method for detecting and treating colon polyps. The results of some studies have shown that removal of adenomas in normal daily practice is associated with a decrease in development of colorectal cancer [4].

Many innovative endoscopic methods such as virtual magnifying chromoendoscopy with flexible spectral imaging color enhancement (FICE) and magnifying chromoendoscopy (as with Indigo carmine and Crystal violet) have been developed to observe the morphological characteristics of the pit pattern and the vascular structure of the colorectal polyps with high accuracy. These three methods still remain their high diagnostic values in Vietnam, a scare-setting resource, but there have been no studies in our area that systematically assessed the accuracy of them in prediction of colorectal polyp pathology. In order to address this issue, we implemented our study.

## 2. Materials and Methods

### 2.1. Patient Selection

#### 2.1.1. Selection Criteria

- Patients agreed to participate in the study
- The patient has clinical symptoms such as abdominal pain, digestive disorders, constipation, and bloody in stools, and polyps were detected during endoscopy
- The patient has no clinical symptoms, but polyps were detected during colonoscopy screening for colorectal cancer
- In patients with clean colon preparation, the degree of colonic cleanliness is assessed according to the Boston scale with a total score of ≥8

#### 2.1.2. Exclusion Criteria:

- Patients with contraindications to total colonoscopy: severe heart failure, severe respiratory failure, shock, and so on
- The patient is indicated for colonoscopy with polyps detected, but the polyp surface has a lot of mucus that cannot be cleaned
- The patient could not biopsy polyps or did not remove all polyps through endoscopy
- Patients with so many colorectal polyps that we could not have enough time to evaluate all polyps by three magnified, stained endoscopic methods. Therefore, we excluded patients with more 4 polyps
- Patients did not agree to participate in the study

From May 2016 to September 2019, a total of 266 patients were eligible to participate in the study with 332 colorectal polyps detected endoscopically at the center for gastrointestinal endoscopy in Bach Mai Hospital, Vietnam.

### 2.2. Protocols

Step 1. White light endoscopy for the entire colon, inserting the endoscope into the Bauhin valve and the ileocecal angle

Step 2. During endoscopic tube withdrawal, close observation to detect colorectal polyps. Polyps will be washed with water

Step 3. Switch to FICE light mode (using the control button on the endoscope). Polyp observations in FICE (channel 4) nonmagnified mode and FICE with 50-150-time magnified mode

Step 4. Switch back to normal light endoscopy mode. Spray 10-20 ml of Indigo carmine dye solution 0.2% to evenly cover the entire polyp surface. Observe the pit pattern with normal light and magnification (50-150 times). Evaluate polyp's pit pattern according to the Kudo's classification. Rinse the polyps with water

Step 5. Spray 5-10 ml crystal violet dye solution 0.05% to evenly coat the polyp surface. Wait 1-2 minutes for the dye to absorb evenly on the polyp. Evaluate the pit pattern of polyps according to the Kudo's classification. For polyps suspected of being cancerous (FICE type V and Kudo type V), all patients were biopsied for pathology. Then a CT scan of the abdomen and chest X-ray for further evaluation

Step 6. Endoscopic polypectomy with biopsy/snare/EMR pliers (with Kudo polyp types II-Vi); ESD when the entire polyp must be removed because it is difficult to perform EMR or surgery is indicated (for polyps classified as Kudo type Vn). In some cases, after EMR/ESD, polyps have histopathological results as cancer that has invaded through the submucosal layer; the patient will have additional surgery

Step 7. After cutting polyps, take specimen into the storage tube. In the case of patients with ESD polyps, the removed lesions will be fixed on a thin sponge with pins before soaking in 10% formol solution. Write down the patient's name and age. Send the specimen to the Department of Histopathology to read the results

Step 8. Compare the results of magnifying chromoendoscopy and histopathology

**Figure 1:**
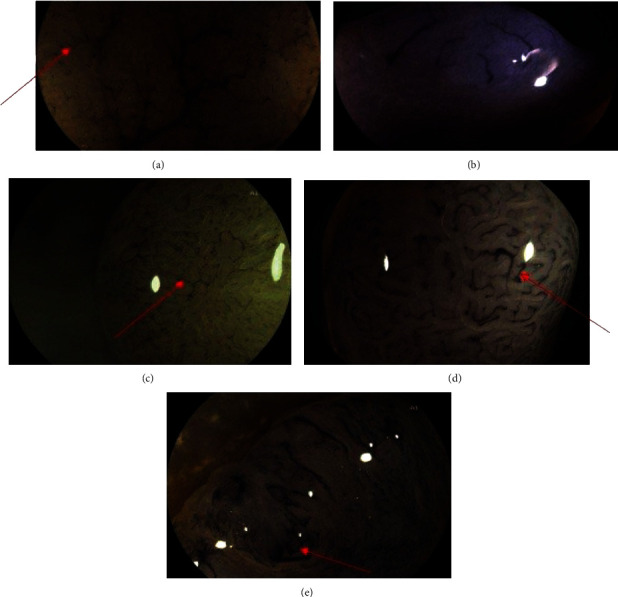
Endoscopic capillary-vessel pattern classification (Teixeira et al.) [5]. (a)Type I: normal pattern composed of thin subepithelial capillary vessels with a linear shape and regular arrangement surrounding the mucosal crypts. (b).Type II: this pattern exhibits hypovascularity or marginal capillaries of a thicker diameter, curved, or straight but uniform, without dilatations, and the pericryptal arrangement is not remarkable. (c).Type III: numerous capillaries of thinner diameter, irregular and tortuous, with frequent point dilatations, and tapering like a spiral shape, showing remarkable periglandular arrangement. (d).Type IV: numerous long, spiral, or straight blood vessels with a thicker diameter, and sparse dilatations, running upright, surrounding the villous glands. (e).Type V: pleomorphism of capillaries and abnormal distribution and arrangement; numerous heterogeneous thick vessels with chaotic arrangement are the predominant.

Capillary-vessel pattern classification by Teixeira (Teixeira's classification) (Figure 1):

- FICE types I and II: corresponding to nonneoplastic polyps
- FICE types III, IV, and V: corresponding to neoplastic polyps

**Figure 2:**
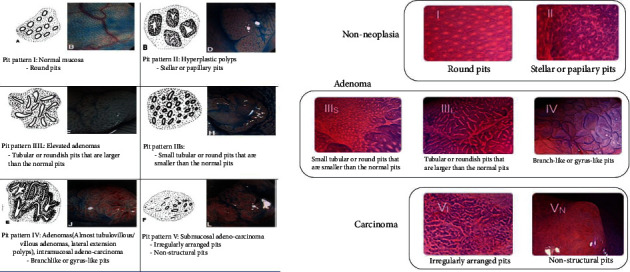
Kudo's pit pattern classification in magnification endoscopy with Indigo carmine and Crystal violet (Huang et al. [6] and Kobayashi et al.) [7].

Pit pattern classification by Kudo (Kudo's classification) (Figure 2):

- Kudo types I and II: corresponding to nonneoplastic polyps
- Kudo types IIIL, IIIs, IV, Vi, and Vn: corresponding to neoplastic polyps

The size of each polyp was estimated using the width of an opened standard biopsy pliers measuring 8 mm as a reference marker. All lesions after endoscopic observation were biopsied and histologically evaluated according to the histopathological classification of the World Health Organization (2010). The results were obtained by the experts at the Department of Pathology, 108 Central Military Hospital (Vietnam).

### 2.3. Statistical Analysis

The data is imported and managed by EPIDATA 3.1 software and is cleaned and analyzed using STATA 12.0 statistical software.

The quantitative variable is presented as the mean number and the standard deviation. The standard variable is presented in the form of frequency and percentage.

Chi-square test and Fisher's exact test evaluate the difference in proportions of more than 1 group. The test results are significant with *p* < 0.05.

The sensitivity, specificity, positive diagnostic value, negative diagnostic value, and accuracy of magnifying chromoendoscopy with Crystal violet, Indigo carmine, and FICE are analyzed.

## 3. Results

By gender, the detection rate of colorectal polyps in male was higher than in female (62.8% versus 37.2%). Ratio of male/female patients = 1.7/1. The mean age of the subjects was 56.4 ± 14.4; min = 17; max = 93. The patients with colorectal polyps are mainly in the age group over 40 years old (83.9%) (Table 1).

In the group of polyps classified as FICE type II, 22/59 (37.3%) were adenomatous polyps, without carcinoma. In the group of polyps classified by FICE type III, 135/154 (87.7%) were adenomas, and 2/154 (1.3%) were carcinomas. In the group of polyps classified by FICE type IV, 69/81 (85.1%) were adenomas, and 12/81 (14.8%) were carcinomas. In the group of polyps classified by FICE type V, 5/38 (13.2%) were adenomas, and 33/38 (86.8%) were carcinomas (Table 2).

Of the polyps classified as Kudo type II, 22/50 (22%) were adenomatous polyps, without carcinoma. Among polyps classified as Kudo type IIIL, 150/166 (90.3%) were adenomas, and 1/166 (0.6%) were carcinomas. Among polyps classified as Kudo type IIIs, 9/11 (81.8%) were adenomatous polyps, and 2/11 (18.2%) were carcinomas. Among polyps classified as Kudo type IV, 53/62 (85.5%) were adenomas, and 9/62 (14.5%) were carcinomas. Among polyps classified as Kudo type V, 8/43 (18.6%) were adenomatous polyps, and 25/43 (81.4%) were carcinomas (Table 3).

In the group of polyps classified as Kudo type II, 8/47 (17%) were adenomatous polyps, without carcinoma. In the Kudo group IIIL, 151/167 (90.4%) were adenoma polyp, and 1/167 (0.6%) were carcinoma. In the Kudo group IIIs, 8/9 (88.9%) were adenomatous polyps, and 1/9 (11.1%) were carcinomas. In the Kudo group type IV, 57/62 (91.9%) were adenomatous polyps, and 5/62 (8.1%) were carcinomas. Among polyps classified as Kudo type Vi, 7/24 (29.2) were adenomas, and 17/24 (80.8%) were carcinomas. Meanwhile, all polyp groups classified as Kudo type Vn have histopathological results as carcinoma (100%) (Table 4). Among the three methods, magnifying chromoendoscopy with Crystal Violet 0.05% has the highest sensitivity and specificity.

Among the three methods, Crystal violet has the highest sensitivity and specificity (97.2% and 72.2%, respectively) in predicting histopathological results of colorectal polyps, compared with Indigo carmine (96.0% and 72.2%) and FICE (92.1% and 68.5%, respectively). Magnifying chromoendoscopy with FICE showed the lowest sensitivity (92.1% compared with 96.0% and 97.1% of Indigo carmine and Crystal violet, respectively). Noticeably, when combining FICE results with Indigo carmine or Crystal violet ones∗ then compare to histopathology, although the sensitivity slightly decreased (91.7% and 91.4%, respectively), the specificity increased remarkably (77.8% and 79.6%, respectively) (Table 5). Kappa value between the endoscopic methods and histopathological results also reported related results: The highest agreement belongs to Crystal violet (kappa = 0.7317, compare with 0.7037 and 0.5843 of Indigo carmine and FICE, respectively) (Table 6).

Interpretation of FICE and Crystal violet result combination: if both results of FICE and Crystal violet showed that polyps were neoplasm, then the polyps will be marked as neoplasm, and the others were nonneoplasm. Interprtation of FICE and Indigo carmine is similar.

## 4. Discussion

The study was conducted on 266 patients aged 17 to 93 years old with the mean age of the subjects was 56.4 ± 14.4 years. Research results showed that men had a higher rate of colorectal polyps than women with the male/female ratio = 1.7/1. Most of the patients in the study were in the group of subjects over 40 years old (83.9%).

In our study, virtual magnifying chromoendoscopy with FICE had sensitivity, specificity, and accuracy in differential diagnosis of neoplastic/nonneoplastic polyps of 92.1%, 68.5%, and 88.3%, respectively. This result is lower than the results of dos Santos et al. in the controlled clinical trial with the corresponding results of 97.8%, 79.3%, and 92.8% [8] but higher than the results of similar study by Togashi et al., with sensitivity, specificity, and accuracy of differential diagnosis of neoplastic/nonneoplastic polyps of 87%, 93%, and 70% [9]. The difference may be due to differences in sample size, subject characteristics, or experience in reading virtual magnifying chromoendoscopy results with FICE. However, previous studies of virtual magnifying chromoendoscopy with FICE mainly focused on evaluation of vascular density or polyp surface morphology, but did not describe the vascular morphology of cancerous polyps. Therefore, in our study, we would like to emphasize the detailed description of the submucosal vascular characteristics of the cancerous lesions with the characteristics of angiogenesis, blood vessels becoming abnormal, dilated, and lost vascular structure. At the same time, the use of the Teixeira classification for virtual magnifying chromoendoscopy with FICE is very accessible and usable in clinical practice for the endoscopist.

Magnifying chromoendoscopy with Indigo carmine 0.2% improves visualization of mucosal orifice morphology and increases accuracy in distinguishing neoplastic/nonneoplastic polyps. According to many studies, the sensitivity of the method has a relatively large difference, ranging from 72% to 99% [8]. According to our study results, the specificity, specificity, and accuracy of magnifying chromoendoscopy with Indigo carmine 0.2% were 96.0%, 72.2%, and 92.2%, respectively. This result has higher sensitivity but lower specificity and accuracy than the results of Kato et al., with sensitivity, specificity, and diagnostic accuracy of 92.3%, 99.8%, and 99.1%, respectively [10]. This difference may be due to differences in different study times and dye properties. However, our results are similar to the study of dos Santos et al. (2010) who investigated the diagnostic value of virtual magnifying chromoendoscopy with FICE and magnifying chromoendoscopy with Indigo carmine 0.2% in 248 lesions. The results of Santos' study showed that the sensitivity, specificity, and accuracy of magnifying chromoendoscopy with Indigo carmine 0.2% (97%, 88.9%, and 94.9%) were higher than those of virtual magnifying chromoendoscopy with FICE (97.8%, 79.3%, and 92.8%) [8]. This shows that magnifying chromoendoscopy with Indigo carmine is a reliable method for predicting with accuracy the histological results of colorectal polyps, better than virtual magnifying chromoendoscopy with FICE. The nature of Indigo carmine is a contrast dye, so it will not be absorbed into the mucosal epithelial cells. The dye will only be deposited in the slits and grooves of the mucosa and allows a clearer display of the lesion boundaries and the polyp surface structure. Therefore, Indigo carmine can be completely cleaned after dyeing. This is distinct from Crystal violet, a dye that is absorbed into the epithelial cells of the mucosa and is difficult to remove with water or conventional washings during endoscopic procedures. Because of this feature, magnifying chromoendoscopy with Indigo carmine remains limited in accurately classifying Kudo type Vi and Vn polyps to predict the extent of submucosal invasion.

The results of our study show that the sensitivity, specificity, and accuracy of neoplastic/nonneoplastic polyp diagnosis by magnifying chromoendoscopy with Crystal violet 0.05% were 97.1%, 77.2%, and 93.1%. This result is similar to the study Matsuda et al. with diagnostic values of 85.6%, 99.4% and 98.8%, respectively [11]. The results of many other studies show that the sensitivity, specificity, and accuracy of the differential diagnosis of neoplastic/nonneoplastic polyps according to the Kudo's pit pattern classification are relatively high. At the same time, the studies have a range of sensitivity (82-94%), specificity (65-93%), and accuracy (80-93%) [12]. In addition, magnifying chromoendoscopy with Crystal violet 0.05% also has advantages in assessing the risk of submucosal invasion of malignant polyps, assessing the relationship between Vi and Vn types (Kudo's classification) and histological results. Pathology showed that, all polyps classified Kudo type Vi, Vn had histopathological results corresponding from high-grade dysplastic polyp to cancer. Specifically, polyps were classified as Kudo type Vi, including 29.2% (7/24) polyps with histopathological results as high-grade dysplasia, 50% (12/24) polyps as intramucosal carcinoma, and 20.8% (5/24) polyps as submucosal carcinoma. Meanwhile, polyps are classified as Kudo type Vn; the majority of polyps are submucosal carcinomas with 78.3% (18/23) polyps; only 21.7% (5/23) are intramucosal carcinomas and no polyps with high-grade dysplasia. Meanwhile, virtual magnifying chromoendoscopy with FICE and magnifying chromoendoscopy with Indigo carmine have limitations in predicting the extent of submucosal invasion with cancerous polyps. Magnifying chromoendoscopy with Crystal violet has been shown to be very good at predicting the invasiveness of cancerous polyps. Then, the appointment of ESD or EMR to remove polyps during surgery will be accurate and appropriate for the patient. Therefore, by magnifying chromoendoscopy with Crystal violet 0.05%, malignant polyps classified as Kudo type Vi (predicted histologically as intramucosal carcinoma) would be indicated for ESD or EMR. Polyps classified as Kudo type Vn (histopathologically predicted submucosal carcinoma) were indicated for surgery.

Among the three methods, Crystal violet has the highest sensitivity and specificity in predicting histopathological results of colorectal polyps. It has also been proved when analyzing Kappa value between the endoscopic methods and histopathology results: The highest agreement belongs to Crystal violet (kappa = 0.7317, compared with 0.7037 and 0.5843 of Indigo carmine and FICE, respectively). We also combine FICE results with those of Indigo carmine or Crystal violet and then compare them to histopathology, although the sensitivity slightly decreased (91.7% and 91.4%, respectively) and the specificity increased remarkably (77.8% and 79.6%, respectively) (Table 5). Therefore, if possible, the practitioners should choose the endoscopic method with the highest diagnostic value for their patients. For example, they can use Crystal violet to achieve high sensitivity or combine Crystal violet with FICE for higher specificity. On the other hand, although magnifying chromoendoscopy with FICE showed the lowest sensitivity (92.1% compare with 96.0% and 97.1% of Indigo carmine and Crystal violet, respectively), the figure still be ideal for endoscopists to initially classify the polyps' pit pattern, especially in scare-setting resources with unavailable high-end diagnostic facility due to limited financial capability, such as in Vietnam.

Magnifying chromoendoscopy with FICE, Indigo carmine, and Crystal violet is a reliable method in the precise prediction of pathogenic tissue results as neoplasm/nonneoplasm polyp. Our research results also contribute to doctors in choosing a suitable diagnostic method based on the level of diagnostic reliability and permissible conditions at a clinical practice facility in Vietnam. Virtual magnifying endoscopy with FICE has the advantage of being more convenient, without a promising color dye spray to apply normally in clinical practice. Magnifying chromoendoscopy with Indigo carmine and Crystal violet has a high precision in predicting pathological tissue results as neoplasm/nonneoplasm polyp. Magnifying chromoendoscopy with Crystal violet will have an advantage for suspected cases of invasive cancer through the colon's mucosal mucosa. This is a decisive factor to help endoscopists determine the best treatment (EMR, ESD, surgery) for patients with colorectal polyps.

With regards to limitations, this study employed the same evaluation sequence, FICE, Indigo carmine, and then Crystal violet, in which the two previous evaluations, in theory, could improve the performance of Crystal violet evaluation. This revealed that each technique should be evaluated by different independent colonoscopists in further studies, with consideration of case control study design to achieve more reliable results.

## 5. Conclusion

Magnifying chromoendoscopy with Crystal violet has the highest sensitivity and accuracy in predicting histopathological results of colorectal polyps. FICE has shown its diagnostic value with reliable sensitivity and should still be a reasonable endoscopic choice for physicians in scare-setting resources regardless its moderate specificity. Physicians should base on their facility and capability to determine an appropriate endoscopy technique.

## Figures and Tables

**Table 1:** General characteristic of subjects.

Characteristics		*n*	%
Gender	Male	167	62.8
	Female	99	37.2
Age	<21	3	1.1
	21–30	11	4.1
	31–40	29	10.9
	41–50	33	12.4
	51–60	83	31.2
	61–70	68	25.6
	>70	39	14.7
	*X* ± *SD*, min, max	56.4 ± 14.4; min = 17; max = 93	

**Table 2:** Relationship between capillary-vessel pattern (Teixeira) classification of magnifying endoscopy with FICE and histopathology results (*n* = 332).

Teixeira classification (FICE)	Histopathology results					Total
	Nonneoplastic polyp	Neoplastic polyp				
	Hyperplastic/juvenile polyp	Adenomas polyp		Carcinoma		
		Low dysplasia	High dysplasia	Intramucosal	Submucosal	
Type II	37 (62.7)	22 (37.3)	0 (0.0)	0 (0.0)	0 (0.0)	59 (17.8)
Type III	17 (11.0)	125 (81.2)	10 (6.5)	2 (1.3)	0 (0.0)	154 (46.4)
Type IV	0 (0.0)	36 (44.4)	33 (40.7)	7 (8.6)	5 (6.2)	81 (24.4)
Type V	0 (0.0)	0 (0.0)	5 (13.2)	13 (34.2)	20 (52.6)	38 (11.4)
Total	54 (16.3)	183 (55.1)	48 (14.5)	22 (6.6)	25 (7.5)	332 (100.0)

**Table 3:** Relationship between Kudo pit pattern classification of magnifying chromoendoscopy with Indigo carmine 0,2% and histopathology results (*n* = 332).

Kudo's classification	Histopathology results					Total
	Nonneoplastic polyp	Neoplastic polyp				
	Hyperplastic/juvenile polyp	Adenomas polyp		Carcinoma		
		Low dysplasia	High dysplasia	Intramucosal	Submucosal	
Type II	39 (78.0)	11 (22.0)	0 (0.0)	0 (0.0)	0 (0.0)	50 (15.1)
Type IIIL	15 (9.1)	142 (85.5)	8 (4.8)	1 (0.6)	0 (0.0)	166 (50.0)
Type IIIS	0 (0.0)	4 (36.4)	5 (45.4)	2 (18.2)	0 (0.0)	11 (3.3)
Type IV	0 (0.0)	25 (40.3)	28 (45.2)	6 (9.7)	3 (4.8)	62 (18.7)
Type V	0 (0.0)	1 (2.3)	7 (16.3)	13 (30.2)	22 (51.2)	43 (12.9)
Total	54 (16.3)	183 (55.1)	48 (14.5)	22 (6.6)	25 (7.5)	332 (100.0)

**Table 4:** Relationship between Kudo pit pattern classification of magnifying chromoendoscopy with Crystal violet and histopathology results (*n* = 332).

Kudo's classification	Histopathology results					Total
	Nonneoplastic polyp	Neoplastic polyp				
	Hyperplastic/juvenile polyp	Adenomas polyp		Carcinoma		
		Low dysplasia	High dysplasia	Intramucosal	Submucosal	
II	39 (83.0)	8 (17.0)	0 (0.0)	0 (0.0)	0 (0.0)	47 (14.2)
IIIL	15 (9.0)	144 (86.2)	7 (4.2)	1 (0.6)	0 (0.0)	167 (50.3)
IIIs	0 (0.0)	5 (55.6)	3 (33.3)	1 (11.1)	0 (0.0)	9 (2.7)
IV	0 (0.0)	26 (41.9)	31 (50.0)	3 (4.8)	2 (3.3)	62 (18.7)
Vi	0 (0.0)	0 (0.0)	7 (29.2)	12 (50.0)	5 (20.8)	24 (7.2)
Vn	0 (0.0)	0 (0.0)	0 (0.0)	5 (21.7)	18 (78.3)	23 (6.9)
Total	54 (16.3)	183 (55.1)	48 (14.5)	22 (6.6)	25 (7.5)	332 (100.0)

**Table 5:** The differential diagnostic value of neoplastic and nonneoplastic polyps of magnifying chromoendoscopy in histological prediction of colorectal polyps.

Methods		Histopathology results			Sensitivity	Specificity	Accuracy
		Neoplasm	Nonneoplasm	Total			
Magnifying chromoendoscopy with FICE	Neoplasm	256	17	273	92.1%	68.5%	88.3%
	Nonneoplasm	22	37	59			
	Total	278	54	332			
Magnifying chromoendoscopy with Indigo carmine 0.2%	Neoplasm	267	15	283	96.0%	72.2%	92.2%
	Nonneoplasm	11	39	50			
	Total	278	54	332			
Magnifying chromoendoscopy with Crystal violet 0.05%	Neoplasm	270	15	285	97.2%	72.2%	93.1%
	Nonneoplasm	8	39	47			
	Total	278	54	332			
Magnifying chromoendoscopy with FICE & Indigo carmine 0.2%	Neoplasm	255	12	267	91.7%	77.8%	89.5%
	Nonneoplasm	23	42	65			
	Total	278	54	332			
Magnifying chromoendoscopy with FICE & Crystal violet 0.05%	Neoplasm	254	11	265	91.4%	79.6%	89.5%
	Nonneoplasm	24	43	67			
	Total	278	54	332			
	Nonneoplasm	12	40	52			
	Total	278	54	332			

**Table 6:** The agreement of differential diagnostic value of neoplastic and nonneoplastic polyps of magnifying chromoendoscopy with histological results.

Methods	Agreement	Expected agreement	Kappa	*p*
Magnifying chromoendoscopy with FICE	88.3%	71.74%	0.5843	<0.001
Magnifying chromoendoscopy with Indigo carmine 0.05%	92.2%	73.57%	0.7037	<0.001
Magnifying chromoendoscopy with Crystal violet 0.05%	93.1%	74.18%	0.7317	<0.001

## Data Availability

The patients' data used to support the findings of this study are available from the corresponding author upon request.

## Supplementary Materials

Supplementary Materials We attached a file named “Supplementary. Manuscript Hindawi. MAGNIFYING CHROMOENDOSCOPY.10.3,” which illustrates images of white light endoscopy and magnifying endoscopy with FICE, images of conventional endoscopy and magnifying chromoendoscopy with Indigo carmine 0.2%, and images of white light endoscopy and magnifying chromoendoscopy with Crystal violet 0.05%.
