# Supplementary material for: Magnifying Chromoendoscopy with Flexible Spectral Imaging Color Enhancement, Indigo Carmine, and Crystal Violet in Predicting the Histopathology of Colorectal Polyps: Diagnostic Value in a Scare-Setting Resource
Source: Gastroenterol Res Pract. 2022 Jul 13;2022:6402904. doi: 10.1155/2022/6402904 (PMC9300359; doi:10.1155/2022/6402904)
Supplement: Supplementary Materials — We attached a file named “Supplementary. Manuscript Hindawi. MAGNIFYING CHROMOENDOSCOPY.10.3,” which illustrates images of white light endoscopy and magnifying endoscopy with FICE, images of conventional endoscopy and magnifying chromoendoscopy with Indigo carmine 0.2%, and images of white light endoscopy and magnifying chromoendoscopy with Crystal violet 0.05%. [file 6402904.f1.docx]

**SUPPLEMENTARY**


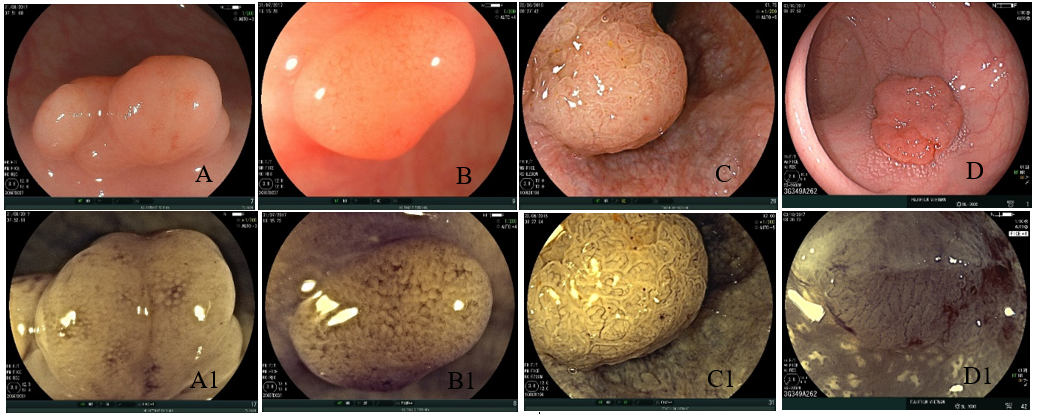


***Figure 1. Images of white light endoscopy and magnifying endoscopy with FICE***

*A-A1: White light endoscopic image (A) and magnifying endoscopy with FICE (A1), capillary pattern classification as FICE type II*

*B-B1: White light endoscopic image (B) and magnifying endoscopy with FICE (B1) capillary pattern classification as FICE type III*

*C-C1: White light endoscopic image (C) và and magnifying endoscopy with FICE (C1) capillary pattern classification as FICE type IV*

*D-D1: White light endoscopic image (D) and magnifying endoscopy with FICE (D1) capillary pattern classification as FICE type V*


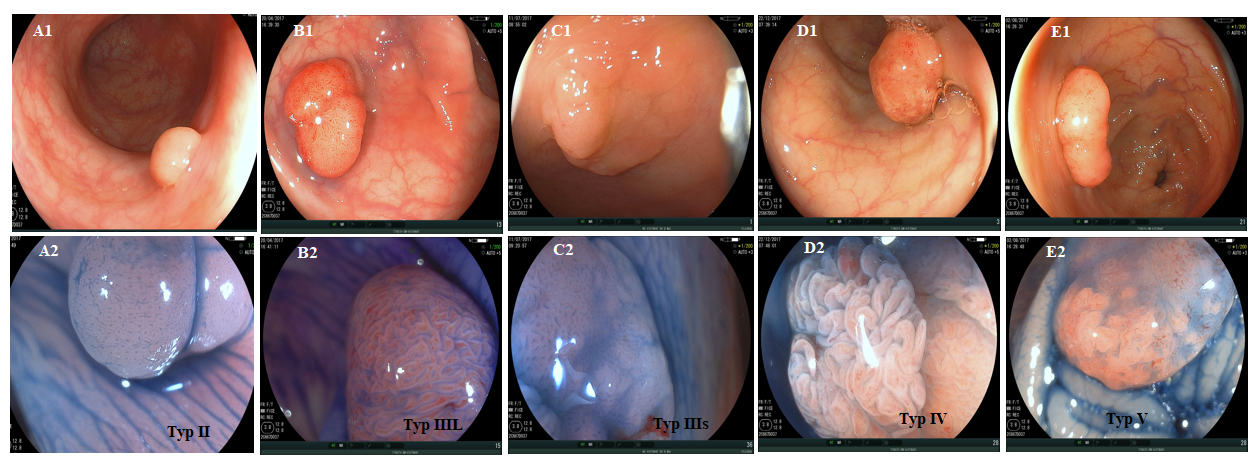


***Figure 2. Images of convential endoscopy and magnifying chromoendoscopy with Indigo carmine 0,2%***

*A1-A2: White light endoscopic image (A1) and magnifying chromoendoscopy with Indigo carmine (A2), classified as Kudo typ II*

*B1-B2: White light endoscopic image (B1) and magnifying chromoendoscopy with Indigo carmine (B2), classified as Kudo type IIIL*

*C1-C2: White light endoscopic image (C1) and magnifying chromoendoscopy with Indigo carmine (C2), classified as Kudo type IIIs*

*D1-D2: White light endoscopic image (D1) and magnifying chromoendoscopy with Indigo carmine (D2), classified as Kudo type IV*

*E1-E2: White light endoscopic image (E1) and magnifying chromoendoscopy with Indigo carmine (E2), classified as Kudo type V.*


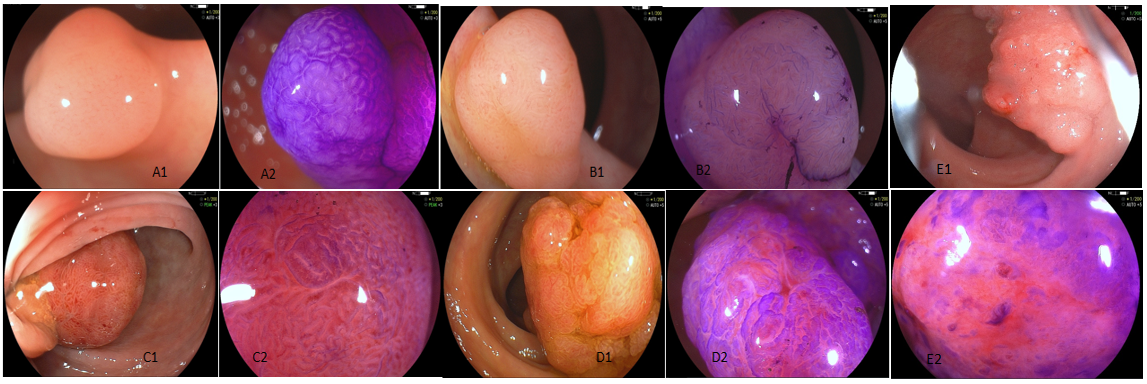


***Figure 3. Images of white light endoscopy and magnifying chromoendoscopy with Crystal violet 0,05%***

*A1-A2: White light endoscopic image (A1) and magnifying chromoendoscopy with Crystal violet (A2), classified as Kudo type II*

*B1-B2: White light endoscopic image (B1) and magnifying chromoendoscopy with Crystal violet (B2), classified as Kudo type IIIL*

*C1-C2: White light endoscopic image (C1) and magnifying chromoendoscopy with Crystal violet (C2), classified as Kudo type IV*

*D1-D2: White light endoscopic image (D1) and magnifying chromoendoscopy with Crystal violet (D2), classified as Kudo type Vi*

*E1-E2: White light endoscopic image (E1) and magnifying chromoendoscopy with Crystal violet (E2), classified as Kudo type Vn*
